# Comparison of ^18^F-FDG PET/CT imaging with different dual time ^18^F-FDG PET/CT with forced diuresis in clinical diagnosis of prostate cancer

**DOI:** 10.1097/MD.0000000000032331

**Published:** 2023-01-13

**Authors:** Longhua Yu, Shiming Huang, Siyu Wu, Jianlan Yue, Liang Yin, Zhichun Lin

**Affiliations:** a Department of Nuclear Medicine, Hospital 971 of The Navy of Chinese PLA, Qingdao, Shandong, China; b Department of Nuclear Medicine, Pingjin Hospital, Characteristic Medical Center of Chinese People’s Armed Police Forces, Tianjin, China; c Department of obstetrics and gynecology, Pingjin Hospital, Characteristic Medical Center of Chinese People’s Armed Police Forces, Tianjin, China.

**Keywords:** ^18^F-FDG, PET/CT, prostate cancer (PCa), SUVmax

## Abstract

The aim of this study was to compare the capability of different dual time (interval 1, 2, 3, or 4 hours) 18F-fluorodeoxyglucose (^18^F-FDG) positron emission tomography/computed tomography (PET/CT) with forced diuresis to diagnose prostate cancer (PCa). A retrospective review of 273 male patients from March 2009 to June 2019, with any focal ^18^F-FDG uptake in the prostate gland during PET/CT imaging. Early PET/CT imaging was performed 60 minutes after FDG injection. Delayed imaging was performed 1 to 4 hours after diuretic injection. For prostate lesions with increased ^18^F-FDG uptake, a spheroid-shaped volume of interest was drawn, including the entire lesion, and the maximum standard uptake value (SUVmax) of the lesion was measured. The SUVmax > 2.5 after delayed imaging and the retention index > 15% were used as the diagnostic criteria for PET/CT in the diagnosis of PCa. Otherwise, it was diagnosed as the benign prostate disease. The final diagnosis was based on histological examination, associated imaging studies, or/and clinical follow-up. The results of inter-group comparison showed that the SUVmax of 1-, 2-, 3-, and 4-hour delayed imaging after diuresis in PCa group was significantly higher than that in control group (*P* < .05), but there was no statistical difference in SUVmax of early imaging between PCa and control group (*P* > .05). And the retention index of PCa group that delayed 1, 2, 3, and 4 hours after diuresis were significantly higher than those of control group, respectively (*P* < .05). The diagnostic sensitivity of imaging delayed 1, 2, 3, and 4 hours after diuresis was 68.8%, 81.2%, 85.7 %, and 71.4%, the specificity was 52.5%, 74.5%, 70.6%, and 65.0%, and the accuracy was respectively 58.2%, 77.4%, 76.4%, and 67.6%, the positive predictive values were 44.0%, 68.9%, 64.3%, and 58.8%, and the negative predictive value were 75.6%, 85.4%, 88.9%, and 76.5%, respectively. ^18^F-FDG PET/CT imaging as an imaging tool lacks certain specificity in the diagnosis of PCa, regardless of whether the imaging is delayed. The main advantage of delayed diuretic imaging in PCa is that it can significantly improve the sensitivity, especially the diagnostic effect delayed 2 hours after diuresis is better.

## 1. Introduction

Prostate cancer (PCa) is a complex disease. It is one of the most common malignant tumors in men (approximately 9.7%). It accounts for 1 to 2% of deaths in men. However, if PCa is detected early and effectively treated, patients with low and moderate risk of recurrence usually have a 10-year overall survival rate as high as 99%.^[1]^ Therefore, early detection and diagnosis of PCa plays an important role in improving the prognosis of patients.

Biopsy is the gold standard for the diagnosis of PCa, but 40 to 50% of patients may miss the diagnosis due to failure to puncture the malignant site or puncture the normal tissue.^[2]^ In addition, biopsy is an invasive examination, which cannot be followed up or repeated examinations, and its acceptance of patients is poor.^[3]^ However, imaging detection includes computed tomography, magnetic resonance imaging (MRI), and ultrasound, but all of them have certain deficiencies.^[4]^

^18^F-Fluorodeoxyglucose (^18^F-FDG) positron emission tomography/computed tomography (PET/CT) is widely used in diagnosis, staging and efficacy monitoring of tumors.^[5]^ In clinical practice, maximum standard uptake value (SUVmax) is mainly used for semi-quantitative evaluation, which can reflect the tumor’s ability to take up the glucose and indirectly reflect its metabolic capacity or biological characteristics.^[6]^ ^18^F-FDG is mainly excreted through the urinary system, and it will interfere the imaging and diagnosis of PCa. However, dual-phase imaging method with forced diuresis protocol in ^18^F-FDG PET/CT can significantly improve the sensitivity of PCa diagnosis by emptying the urine. But there is no consensus on the specific delay time.^[7]^ Some studies showed that the delay time should be at least 2 hours after the injection of ^18^F-FDG, and the SUVmax after delayed imaging of of malignant tumor can be increased by 80 to 90%. And the uptake may not be further increased 4 to 5 hours after ^18^F-FDG is injected.^[8]^ Therefore, in order to explore the influence of dual-phase imaging for PCa and the best delay time after diuresis, this study intended to compare the SUVmax values after 1, 2, 3, and 4 hours after forced diuresis protocol.

## 2. Material and methods

### 2.1. Patient selection

This study was conducted with the approval of Ethics Committee of Characteristic Medical Center of PAP. A retrospective review of 273 male patients from March 2009 to June 2019, with any focal ^18^F-FDG uptake in the prostate gland during PET/CT imaging.

Inclusion criteria include no history of PCa or other malignangt tumors, and no treatment for prostate; whole-body ^18^F-FDG PET/CT scan was performed; and the pathological examination results (including postoperative or needle biopsy) clearly indicate that PCa were included in the PCa group. Patients who were excluded from PCa by pathological examination, or by other imaging tests and had not been diagnosed with PCa after at least 2 years of follow-up were included in the prostate benign lesion group (control group).

### 2.2. ^18^F-FDG-PET/CT image acquisition

^18^F-FDG PET/CT imaging were performed using a Discovery ste16 scanner (General Electric Medical Systems, America). All examinees underwent ^18^F-FDG PET/CT imaging 60 minutes after intravenous administration of 3.70 to 5.55 MBq/kg of ^18^F-FDG. Fasting blood glucose was controlled below 10 mmol/L before ^18^F-FDG was injected.

After urinating, initial PET/CT scan covered the head, neck, thorax, abdomen, pelvis, and thigh. By using a 16 detector row scanner, CT images were acquired with the following parameters: CT tube voltage 120 kV, tube current 150 mA, 3.75 mm slice thickness, pitch 1.375, matrix 512 × 512, field of view 50 cm, and PET scanning 3 min/bed. After the data were collected by scanning, the computer uses CT data to automatically attenuate the PET image. The data was sent to the AW4.4 workstation and iteratively reconstructed using the ordered subset maximum expected value method.

Early PET/CT imaging was performed 60 minutes after FDG injection. After the early scan, furosemide was injected. Delayed ^18^F-FDG PET/CT scan after furosemide injection (1, 2, 3, and 4 hours later) only covered the pelvis and abdomen.

### 2.3. Image analysis

Two fellowship-trained board-certified nuclear medicine physicians, with >20 years of experience in the interpretation of PET/CT, reviewed PET/CT images and measured the PET/CT parameters of the prostate lesion.

Lesions that did not represent physiological entities were selected for further evaluation with visually discernible ^18^F-FDG uptake and were associated with distinct correlation on CT. For prostate lesions with increased ^18^F-FDG uptake, a spheroid-shaped volume of interest was drawn, including the entire lesion, and the SUVmax of the lesion was measured. First, the PET/CT images in the initial phase were evaluated and a preliminary report was issued. Next, delayed PET/CT imaging were performed, and then revised the preliminary reports as needed to form the final reports by evaluating the delayed-phase PET/CT images.

The past literature has shown differences between physiological and pathological lesions. Specifically, physiological lesions could change location or shape on delayed images, while pathological lesions remained visually unchanged.^[7]^ Therefore, when the lesion signals detected on the early images changed in location or shape on the delayed-phase images, they were regarded as a physiological uptake. Whereas, the lesions were detected only on the delayed images were considered abnormal lesions.

For ^18^F-FDG, the retention index (RI) from early (SUVmax–E) to delayed phase (SUVmax–D) were calculated by the following formula: RI (%) = (SUVmax–D – SUVmax–E)/ SUVmax–E × 100%. The SUVmax > 2.5 after delayed imaging and the RI > 15% were used as the diagnostic criteria for PET/CT in the diagnosis of PCa. Otherwise, it was diagnosed as the benign prostate disease.

### 2.4. Statistical methods

The *t* test was used to determine if there was a significant difference between the SUVmax and RI values of the PCa and control groups. In the PCa group or the control group, the multiple groups of different delayed imaging time after diuresis (1, 2, 3, and 4 hours) were compared by one-way analysis of variance. The specificity, sensitivity, accuracy, negative predictive value and positive predictive value for PCa were calculated using pathological results as a reference standard. A *P* value <0.05 was considered statistically significant in each analysis. All statistical tests were performed using SPSS Statistics 17.0 (SPSS Inc., Chicago, IL).

## 3. Results

The median age of patients with PCa was 67.32 ± 13.61 years (age range, 49.0–89.0 years). And there were 32, 38, 21, and 14 patients in the delayed 1-, 2-, 3-, and 4-hour group after diuresis, respectively. In control group, the median age of patients with benign lesions was (mean age 68.47 ± 12.58 years, range, 46–88 years). And there were 59, 55, 34, and 20 patients in the delayed 1-, 2-, 3-, and 4-hour group after diuresis, respectively.

**Figure 1.**
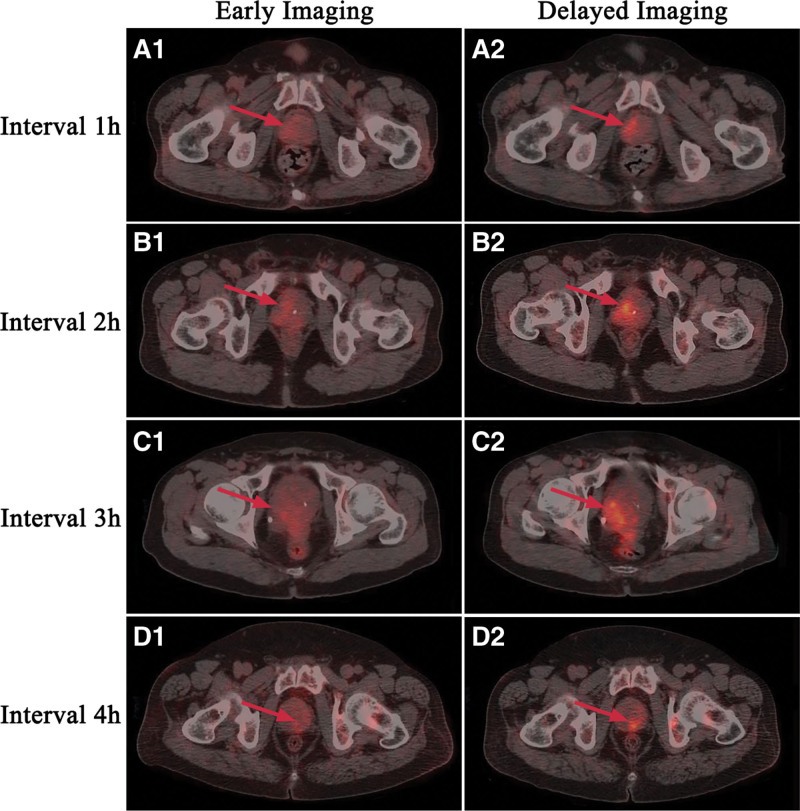
PET/CT imaging of patients with prostate malignant lesions early imaging and delayed imaging after dieresis. (A) The SUVmax of early imaging and delayed 1-hour imaging after diuresis were 3.8 and 4.9, respectively. (B) The SUVmax of early imaging and delayed 2-hour imaging after diuresis were 4.0 and 8.7, respectively. (C) The SUVmax of early imaging and delayed 3-hour imaging after diuresis were 4.1 and 6.7, respectively. (D) The SUVmax of early imaging and delayed 4-hour imaging after diuresis were 3.7 and 5.9, respectively. PET/CT = positron emission tomography/computed tomography, SUVmax = maximum standard uptake value.

In PCa group, the SUVmax of early imaging was 4.43 ± 1.24, 4.56 ± 1.88, 4.62 ± 1.56, and 4.42 ± 1.62 in the group of 1, 2, 3, and 4 hours delayed after diuresis, respectively. And the SUVmax after diuresis were 5.59 ± 2.01, 7.24 ± 2.85, 7.18 ± 2.92, and 5.78 ± 2.97, in the group of 1, 2, 3, and 4 hours delayed after diuresis, respectively. The *t* test results showed that the SUVmax of 1, 2, 3, and 4 hours delayed after diuresis were significantly higher than early imaging (*P* < .05), respectively (Fig. 1). The results of one-way analysis of variance showed that there was no significant difference in the SUVmax of early imaging between the groups of 1, 2, 3, and 4 hours delayed after diuresis (*P* > .05). The comparison of the SUVmax between the groups of 1, 2, 3, and 4 hours delayed after diuresis showed that 2- and 3-hour group were significantly >1 hour group (*P* < .05), the others had no statistical difference between groups (*P* > .05) (Table 1).

**Table 1:** Comparison of SUVmax of between the early imaging and 1-, 2-, 3-, and 4-hour delayed imaging after diuresis (‾x ± s).

	Interval 1 h		Interval 2 h		Interval 3 h		Interval 4 h	
	Early imaging	Delayed imaging	Early imaging	Delayed imaging	Early imaging	Delayed imaging	Early imaging	Delayed imaging
Control group	4.49 ± 1.83	4.73 ± 1.79	4.52 ± 1.63	4.75 ± 2.20	4.47 ± 1.43	4.66 ± 1.88	4.57 ± 1.72	4.41 ± 1.64
PCa group	4.43 ± 1.24	5.59 ± 2.01*^,^†	4.56 ± 1.88	7.24 ± 2.85*^,^†	4.62 ± 1.56	7.18 ± 2.92*^,^†	4.42 ± 1.62	5.78 ± 2.97*

* There were statistically significant differences between PCa group and control group in 1h, 2h, 3h, and 4h delayed group, respectively, *P* < .05. †There were statistically significant differences between early imaging and delayed imaging (1h group, 2h group, 3h group, and 4h group) after diuresis, respectively, *P* < .05.

PCa = prostate cancer, SUVmax = maximum standard uptake value.

In control group, the SUVmax before diuresis was 4.49 ± 1.83, 4.52 ± 1.63, 4.47 ± 1.43, and 4.57 ± 1.72 in the group of 1, 2, 3, and 4 hours delayed after diuresis, respectively. And the SUVmax after diuresis were 4.73 ± 1.79, 4.75 ± 2.20, 4.66 ± 1.88, and 4.41 ± 1.64 in the group of 1, 2, 3, and 4 hours delayed after diuresis, respectively. The *t* test results showed that the SUVmax of 1-, 2-, 3-, and 4-hour delayed imaging after diuresis were significantly higher than early imaging (*P* < .05), respectively. The results of one-way analysis of variance showed no significant difference in SUVmax among all groups (*P* > .05). Comparison of SUVmax between the groups of 1, 2, 3, and 4 hours delayed after diuresis showed no statistical difference among all groups (*P* > .05).

The results of inter-group comparison showed that the SUVmax of 1-, 2-, 3-, and 4-hour delayed imaging after diuresis in PCa group was significantly higher than that in control group (*P* < .05), but there was no statistical difference in SUVmax of early imaging between PCa and control group (*P* > .05).

The RI of PCa group that delayed 1, 2, 3, and 4 hours after diuresis were 28.6% ± 11.8%, 46.6% ± 18.0%, 55.2% ± 21.9%, and 34.6.3% ± 15.2%, respectively, which were significantly higher than those of control group (5.9% ± 5.7%, 6.7% ± 7.1%, 4.1% ± 6.2%, and −4.5% ± 5.9%, respectively) (*P* < .05).

The diagnostic sensitivity of imaging delayed 1, 2, 3, and 4 hours after diuresis was 68.8%, 81.2%, 85.7 %, and 71.4%, the specificity was 52.5%, 74.5%, 70.6%, and 65.0%, and the accuracy was respectively 58.2%, 77.4%, 76.4%, and 67.6%, the positive predictive values were 44.0%, 68.9%, 64.3%, and 58.8%, and the negative predictive values were 75.6%, 85.4%, 88.9%, and 76.5%.

## 4. Discussion

PCa is difficult to be diagnosed early, due to its slow progress and lack of specificity in clinical symptoms. Approximately 70% patients have developed to the terminal stage at the time of initial diagnosis and have lost the best time for first-line treatment, with poor prognosis. However, the early diagnosis and standardized treatment of PCa patients could significantly improve the prognosis.^[9]^

At present, the most commonly used method of PCa screening is blood prostate-specific antigen detection, but it could only be used as the initial screening of PCa, has no clinical value for the stage of PCa.^[10]^ Digital rectal examination is also one of the most economical, direct, and safe methods for PCa screening. However, only 1/3 the patients of suspected patients with rectal examination were eventually diagnosed with PCa, and some patients were already in the terminal stage after further examination.^[11]^ MRI has a high sensitivity for the diagnosis of PCa, but studies have shown that MRI can still miss 15 to 20% of PCa, and the specificity of MRI is low, of which about 50% of biopsies are benign.^[12]^ PET/CT, as a whole body examination method, has important clinical value in the diagnosis and staging of tumors.

Currently, a variety of radioactive imaging agents have been used for the diagnosis of PCa, such as ^68^Ga-prostate specific membrane antigen, and ^11^C-choline, ^11^C-acetate.^[13–15]^ However, the imaging agents mentioned above can only be used in a few hospitals with radionuclide accelerators, and most department of nuclear medicine do not have accelerators and cannot use these imaging agents.^[16]^ Based on its good physical and chemical properties, ^18^F-FDG is currently the most widely used PET/CT imaging agent in the field of nuclear medicine, and delayed imaging has certain clinical value for the differential diagnosis of tumors.^[7]^

The basic principle of PET/CT delayed imaging is to take the advantage of changes in tissue ^18^F-FDG uptake and clearance during the time interval between early imaging and delayed imaging. During delaying imaging, tumor tissue showed stable or increased uptake of ^18^F-FDG over time due to increased proliferation rate of malignant tumors cells and increased expression of hexokinase II and glucose transporter-1. In contrast, ^18^F-FDG was continuously cleared from normal tissue. In most normal tissues, ^18^F-FDG uptake is significantly reduced 1 hour to 3 hours after the ^18^F-FDG injection, thus increasing the contrast between lesions and background and facilitating the accurate identification of lesions. In addition, a longer distribution time after diuresis can significantly improve the clearance of ^18^F-FDG in blood and urinary tract, which is conductive to further reduce the activity of background and improve prostate imaging. Therefore, delayed imaging after diuresis can improve the sensitivity and specificity of PET/CT for PCa diagnosis.^[7]^ Nowadays, there is still no special optimal delay time recommended.

In most studies, there was a significant difference in delayed imaging time. Some studies have a very short time interval between early imaging and delayed imaging (about 30–45 minutes),^[17,18]^ while others had a longer interval (120–217 minutes).^[7,19,20]^ In addition, there were also significant difference in early imaging time of some studies, and the early imaging time and delayed time of different studies might overlap.^[21,22]^ The difference of imaging time might be the main reasons for the inconsistent results.

The relatively objective index of ^18^F-FDG PET/CT imaging is semi-quantitative analysis by SUVmax. And the criteria for delayed PET/CT imaging for the diagnosis of tumors include: SUVmax > 2.5 or (and) increased SUVmax of delayed imaging (relative to early imaging).^[23]^ The study showed that SUVmax increased after delayed imaging compared with early imaging, which often indicated the possibility of malignant tumor, and its positive predictive value was 40.3%. While the positive predictive value of SUVmax remains basically unchanged or decreased was 18.9% and 20.5%, respectively.^[24]^ In addition, there are significant differences in the RI threshold used to judge malignant tumors in different studies. Although the use of SUVmax or RI alone will increase the sensitivity of diagnosis, it will also reduce the specificity.^[19,25]^ And the SUVmax > 2.5 of delayed imaging combined with RI was considered to be a better evaluation standard, which can further improve the specificity of diagnosis.^[26]^

Because the benign lesions of prostate (such as prostatitis) may also have RI > 0%, and Mortensen MA et al^[27]^ showed that the average RI of PCa after delayed imaging was 32%. Therefore, in order to explore the optimal delayed imaging time after diuresis of PCa and to improve the accuracy of diagnosis, SUVmax > 2.5 and RI > 15% were used as the diagnosis criteria for PCa in this study. The changes of SUVmax at 1, 2, 3, and 4 hours after diuresis and its diagnostic efficacy for PCa were compared.

The resulted showed that the SUVmax of PCa at 1, 2, 3, and 4 hours after diuresis were significantly higher than the control group. For the early imaging of PCa lesions with unclear or inconspicuous display before diuresis, delayed diuretic imaging may make the lesions more clear or obvious, which is more conducive to the diagnosis of PCa, and can also provide reference for clinical puncture biopsy. In the PCa group, the SUVmax of 2- and 3-hour interval was significantly higher than that of 1 hour, indicating that the SUVmax of 2-hour and 3-hour interval was the most obvious increase.

In terms of diagnostic efficiency, the most sensitive is the interval of 3 hours, the most specific is the interval of 2 hours, and the most accurate is the interval of 2 hours. In addition, considering the waiting time of patients, delayed 2-hour examination after diuresis may be the best choice for ^18^F-FDG PET/CT imaging examination for PCa patients. The results of this study were consistent with the delayed imaging time recommended by Cheng G,^[28]^ that is, delayed imaging performed 2 hours after diuresis was the most ideal.

However, in the results of this study, the specificity of different delay times after diuresis is not particularly high, which may be related to the influence of the ability of PCa to take up glucose by many other factors: ^18^F-FDG is mainly excreted through the urinary system. After diuresis, a small amount of ^18^F-FDG may still accumulate in the urethra near the bladder and prostate, which may cover part of pathological uptake of PCa. The low expression of glucose transporter-1 on the surface of PCa tumor cells results in low ^18^F-FDG uptake by PCa. The uptake of ^18^F-FDG in some smaller PCa lesions is not very obvious, so it will cause certain false positives or false negative.^[29]^ Benign diseases such as prostatic hyperplasia and prostatitis can take up ^18^F-FDG. In addition, normal prostate can also increase the uptake of 18F-FDG, which can be shown as diffuse ^18^F-FDG concentration, with SUVmax of 1.6 to 3.4.^[27]^ Most benign lesions show a decrease in ^18^F-FDG uptake on delayed imaging, but uptake may also increase over time in some infectious and noninfectious inflammatory lesions. Therefore, the uptake of ^18^F-FDG in PCa lacks certain specificity, which will reduce its diagnostic specificity. In addition, due to differences in tumor cells (tumor genotype, degree of differentiation) or environment (interstitial matrix, vascular distribution, immune status, etc.), there are also heterogeneity among different histological subtypes of PCa. And there may be heterogeneity between different patients of the same tumor subtype, even between different lesions of the same patient. The existence of heterogeneity will affect the uptake of ^18^F-FDG in both early and delayed PET/CT imaging, and ultimately affect its diagnostic.

There are some shortcomings in this study: limited cases were included in the group of PCa group and the control group could not all be diagnosed by pathological examination. A small number of patients were confirmed by follow-up, and some early PCa may exist but not found. Even patients whose puncture results were benign lesions, it may be missed because of not puncturing the malignant tissue. In addition, delayed imaging after diuresis also has some disadvantages, such as patients may receive more radiation exposure and need to wait for a longer time. Although the effective systemic radiation dose will not cause acute radiation damage to the patient, the potential risks are unclear.^[30]^

## 5. Conclusion

In summary, ^18^F-FDG PET/CT imaging as an imaging tool lacks certain specificity in the diagnosis of PCa, regardless of whether the imaging is delayed. However, the main advantage of delayed diuretic imaging in PCa is that it can significantly improve the sensitivity, especially the diagnostic effect of delayed 2 hours after diuresis is better. Therefore, delayed diuretic imaging should be applied in clinical practice to optimize its diagnostic performance. However, delayed imaging may not be of additional clinical value for patients whose diagnosis can be confirmed by early imaging, for example, in patients without suspected metastasis, but pathologically confirmed early tumor before PET/CT examination, or with obvious signs of distant metastasis.

## Author contributions

**Methodology:** Jianlan Yue, Liang Yin

**Writing – original draft:** Shiming Huang.

**Writing – review & editing:** Longhua Yu, Siyu Wu, Zhichun Lin.
